# A small molecule modulating monounsaturated fatty acids and Wnt signaling confers maintenance to induced pluripotent stem cells against endodermal differentiation

**DOI:** 10.1186/s13287-021-02617-x

**Published:** 2021-10-21

**Authors:** Vahid Hosseini, Ashkan Kalantary-Charvadeh, Maryam Hajikarami, Parisa Fayyazpour, Reza Rahbarghazi, Mehdi Totonchi, Masoud Darabi

**Affiliations:** 1 Student Research Committee, Tabriz University of Medical Sciences, 5166615573 Tabriz, Iran; 2 Stem Cell Research Center, Tabriz University of Medical Sciences, 516615731 Tabriz, Iran; 3 Department of Biochemistry and Clinical Laboratories, Faculty of Medicine, Tabriz University of Medical Sciences, Tabriz, Iran; 4 Department of Clinical Biochemistry, Faculty of Medicine, Hamadan University of Medical Sciences, Hamadan, Iran; 5 Department of Stem Cells and Developmental Biology, Cell Science Research Center, Royan Institute for Stem Cell Biology and Technology, ACECR, Tehran, Iran; 6 Department of Applied Cell Sciences, Faculty of Advanced Medical Sciences, Tabriz University of Medical Sciences, Tabriz, Iran; 7 Department of Genetics, Reproductive Biomedicine Research Center, Royan Institute for Reproductive Biomedicine, ACECR, Tehran, Iran; 8 Department of Internal Medicine IV, Heidelberg University Hospital, Heidelberg, Germany

**Keywords:** Germ layers, Desaturation, Pluripotent stem cells, Post-translational modification, Wnt signaling pathway, Wnt3a protein

## Abstract

**Background:**

Stearoyl-coenzyme A desaturase 1 (SCD1) is required for de novo synthesis of fatty acids. Through the fatty acid acylation process, this enzyme orchestrates post-translational modifications to proteins involved in cell development and differentiation. In this study, we used biochemical methods, immunostaining, and covalent labeling to evaluate whether a small molecule modulating unsaturated fatty acids can influence the early endodermal differentiation of human-induced pluripotent stem cells (iPSCs).

**Methods:**

The hiPSCs were cultured in an endoderm-inducing medium containing activin A and defined fetal bovine serum in the presence of an SCD1 inhibitor at different time points. The cell cycles and the yields of the three germ layers (endoderm, mesoderm, and ectoderm) were assessed using flow cytometry. The expression of endoderm and pluripotency markers and the expressions of Wnt signaling pathway proteins were assessed using western blotting and RT-PCR. Total protein acylation was evaluated using a click chemistry reaction.

**Results:**

When SCD1 was inhibited on the first day, the population of cells with endodermal features decreased at the end of differentiation. Moreover, early SCD1 inhibition preserved the properties of hiPSCs, preventing their shift toward mesodermal or ectodermal lineage. Also, first-day-only treatment of cells with the SCD1 inhibitor decreased β-catenin gene expression and the intensity of fluorescent emission in the click chemistry assay. The cells were effectively rescued from these effects by cotreatment with oleate. Late treatment with the inhibitor in the two subsequent days of endoderm induction did not have any significant effects on endoderm-specific markers or fluorescent intensity. Reproducible results were also obtained with human embryonic stem cells.

**Conclusion:**

The small molecule SCD1 inhibitor attenuates the Wnt/β-catenin signaling pathway, conferring the maintenance of hiPSCs by opposing the initiation of endoderm differentiation. The immediate requirement for SCD1 activity in the endoderm commitment of pluripotent stem cells may be of importance in disorders of endoderm-derived organs and dysregulated metabolism.

The schematic representation of the study design and main results. Activin A induces endoderm features through Smad2/3/4 and increases the expression of SCD1. SCD1 can produce MUFAs and subsequently modify the Wnt molecules. MUFA acylated/activated Wnts are secreted to interact with corresponding receptors on the target cells. β-catenin accumulates in the cytoplasm and is translocated into the nucleus after the interaction of Wnt with the receptor. Then, β-catenin increases the expression of the endoderm markers Sox17 and CXCR4.
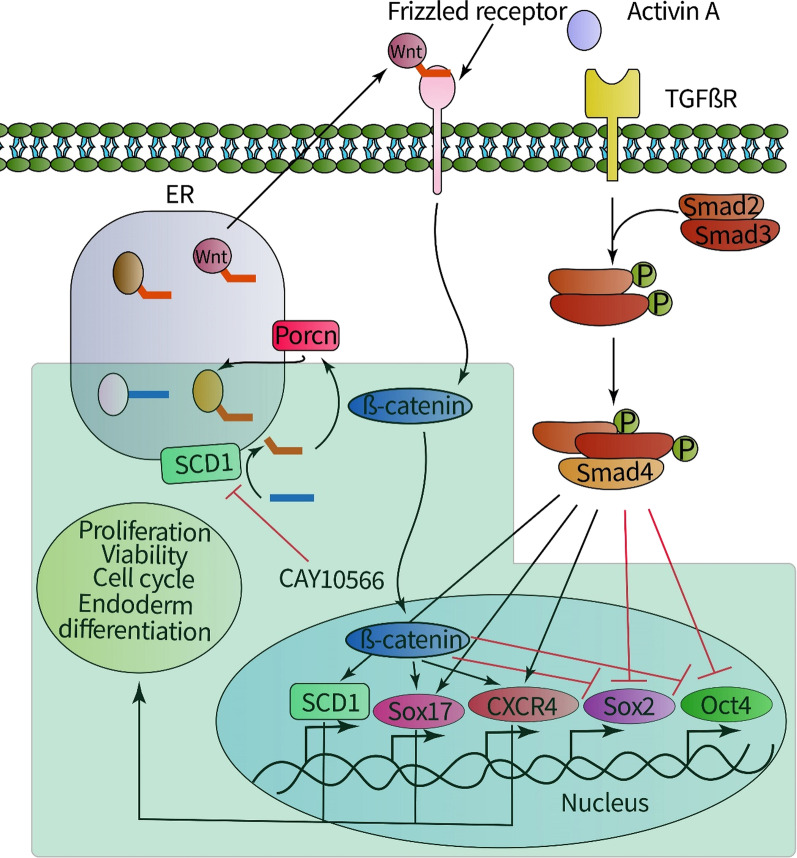

**Supplementary Information:**

The online version contains supplementary material available at 10.1186/s13287-021-02617-x.

## Introduction

Human-induced pluripotent stem cells (hiPSCs) are directly generated from adult somatic cells by reprogramming procedure. It has been suggested that hiPSCs have a great potential to commit into endodermal, mesodermal, and ectodermal cell lineages [1]. The existence of unique pluripotencies, high proliferation rates, lack of unwanted immunological reactions, and lack of ethical concerns make hiPSCs valuable biological tools in the area of regenerative medicine [2]. Furthermore, hiPSCs can be used in biomedical science for applications such as drug screening, finding the genetic basis of diseases, and investigating molecular mechanisms that regulate differentiation [3–6].

Monounsaturated fatty acids (MUFAs) are essential precursors for building cell structures and bioactive lipids [7]. Of note, the endoplasmic reticulum enzyme stearoyl-coenzyme A desaturase 1 (SCD1) converts saturated fatty acids (SFAs) into MUFAs by adding a double bond between carbons 9 and 10, hence regulating the ratio of MUFAs to SFAs in cells [8, 9]. Wnt ligand proteins are the most well-known biomolecules to undergo post-translational modification by addition of MUFAs. This process involves Porcupine (Porcn) catalytic activity, which appends MUFAs, particularly palmitoleate, onto the Ser209 residue of Wnt proteins [10]. It has been indicated that there is a close association between Wnt proteins and MUFAs. For instance, Wnt protein activation, intra-cellular and extra-cellular transportation, and binding to cell surface receptors are dependent on MUFA residues. In previous studies, the critical role of Wnt3a, as the main member of the Wnt family, has been documented in the niche of stem cells [11–13]. Like other members of the Wnt cascade, Wnt3a can transfer signaling to downstream effectors, such as the β-catenin/canonical pathway after MUFA-mediated interaction with cell surface receptors. The synergic effect of Wnt3a and activin A was shown in the differentiation of endoderm-derived organs [14, 15]. In human embryonic stem cells (hESCs) and in induced pluripotent stem cells of mice (miPSCs), the activity of SCD1 supports cell growth and survival [16]. The in vitro suppression of SCD1 can lead to the selective elimination of pluripotent cells [17]. Interestingly, the injection of miPSCs and hESCs pre-treated with an SCD1 inhibitor reduced anaplastic changes and teratoma formation in immunosuppressed mice. Moreover, this treatment can improve stem cell safety after cardiac transplantation [17, 18]. As a correlate, these findings confirm the crucial role of SCD1 in stem cell bioactivity and functional fate acquisition. High intracellular rates of SCD1 have been reported in endoderm-derived tissues, such as liver and pancreas tissue. Similarly, SCD1 content is also relatively high in some non-endodermal tissues such as adipose tissue, indicating SCD1’s tissue- and lineage-dependent activity [19, 20]. Furthermore, SCD1 activity in stem cells is relevant to the characteristics of adipogenic differentiation [21]. Previously, our research group showed the importance of SCD1 products in the hepatic differentiation of hiPSCs [22], which coincided with in vivo data [23]. Considering these findings, one could suggest that SCD1 products not only can enter the metabolic pathways but are also potentially involved in the signaling pathways related to cell differentiation.

There is no report to date assessing the kinetic effect of the small molecules targeting SCD1 during the endodermal differentiation of pluripotent stem cells (PSCs). In this study, we used biochemical methods, acylation assay, and immunostaining to evaluate whether the dynamics of SCD1 activity are important in the differentiation of hiPSCs into endodermal lineages. According to the results, small molecule SCD1 inhibitors can support the maintenance of hiPSCs by postponing endodermal differentiation.

## Materials and methods

### Materials

All cell culture materials were obtained from Gibco (USA) unless otherwise specified.

### Primary mouse embryonic fibroblasts isolation

Primary mouse embryonic fibroblasts (MEFs) were isolated by a mechanical technique and characterized as described in our previous study [24]. In short, embryonic fibroblasts with the passage number 1–5 were commonly proliferated in a culture flask pre-coated with 0.2% gelatin (Sigma, Germany) containing 10 ml of Dulbecco’s minimum eagle’s medium (DMEM; Gibco, USA) and 10% fetal bovine serum (FBS). Before stem cell culture, MEFs were treated with 10 µg/ml Mitomycin C (Abcam, UK) for 3 h to inhibit cell proliferation.

### Stem cell culture and expansion

The established normal R1-hiPSC and hESC XX Royan H1 cell lines [25] were used in this study. The PSCs were characterized by stemness gene expression [25]. The PSCs culture media was composed of 75% DMEM/F12 supplemented with 20% knockout serum replacement, 1% non-essential amino acids, 2 mM L-glutamine (Sigma, Germany), 0.1% β-mercaptoethanol (Merck, Germany), 1% insulin/transferrin/selenium solution, and 12 ng/ml basic human recombinant FGF (b-FGF) (Royan, Iran). The standard cultures were split at the ratio of 1:5 every 7–10 days.

### Endoderm differentiation

Upon 70–80 confluence, hiPSCs were detached using 1 mg/ml Collagenase Type IV (Stem Cell Technologies, Canada) and dissociated into single cells by gentle pipetting. The cells were washed with phosphate-buffered saline (PBS) and suspended in fresh ESCs culture media containing 100 ng/ml of b-FGF, and then transferred on Matrigel- (Sigma, Germany) coated 60 mm plates. The next day, the medium was replaced with the differentiation medium containing RPMI-1640 with 100 ng/ml activin A (BioLegend, USA) with varying concentrations of defined-FBS (D-FBS). The concentrations of D-FBS were set to 0% (Day 0), 0.2% (Day 1), and 2% (Day 2 and 3). The cells were harvested and used for analyses at the fourth 24 h (Day 4) [26].

### Targeting SCD1 by small molecule inhibitor

Chemical inhibition of SCD1 was performed using the specific inhibitor of SCD1 CAY10566 (Cayman Chemical, USA). Cells were treated with SCD1 inhibitor for 24 h at Days 0, 1, or 2 of the differentiation procedure. In our previous study [22], the possible toxicity of the inhibitor was determined using Trypan Blue exclusion and sulforhodamine B assays. Based on that, possible toxicity was excluded using 25 nmol/l CAY10566 [22]. The rescue experiments were performed using 50 µM oleate.

### Evaluation of cell viability

An annexin V-FITC/propidium iodide (PI) apoptosis detection kit (eBioscience, USA) was used for the apoptosis/necrosis analysis. After completion of the endodermal differentiation procedure, cells were detached with collagenase IV and washed once with PBS. The cells were suspended in the binding buffer (1X), incubated with 5 µl annexin-V for 10 min, and washed once with binding buffer. The cells were re-suspended again in 200 µl of binding buffer followed by the addition of 10 µl of propidium iodide. The percentage of apoptosis and necrosis was determined using a flow cytometer (Miltenyi Biotec, USA) and results were analyzed using flow Jo 7.6.1 software (Tree Star, USA).

### Morphological examination

The morphological alteration was visually monitored using a Cytation 5 Cell Imaging Multi-Mode Reader system (BioTek, USA). Differentiating cells were imaged every 24 h for consecutive 4 days in the absence or presence of CAY10566.

### Gene expression assay

Total RNA extraction was carried out using an RNA extraction kit (TaKaRa, Japan) following the instructions provided by the manufacturer. The quantity and quality of extracted RNA were checked using a spectrophotometer (NanoDrop Technologies, USA) and running on 1.5% agarose gel, respectively. The complementary DNA (cDNA) required for real-time PCR was synthesized with a cDNA synthesis kit (Roche, UK). Real-time PCR was performed with SYBR Green PCR master mix (Yekta Tajhiz Azma, Iran) on a MIC real-time PCR system (BioMolecular Systems, Australia). The primer sequences used for Sox2, Oct4, Sox17, and CXCR4 expression assay are listed in Additional file 1: Table S1. Relative gene expression was normalized against the expression level of GAPDH as a reference gene for each sample and all alterations were expressed as fold-changes relative to the mock.

### Surface markers analysis

The pellet of disassociated cells was resuspended in 4% paraformaldehyde to fix the cells by a 30 min incubation at 4 °C. Cells were then resuspended in PBS containing 1% BSA as a staining buffer and incubated for 20 min at 4 °C. Then, cells were incubated with Alexa Fluor 488-conjugated CXCR4 (R&D, USA) and PE-conjugated KDR (R&D, USA), Alexa Fluor 488-conjugated SSEA-3, (eBioscience, USA), or PE-conjugated NCAM antibodies (BioLegend, USA) at 4 °C for 1 h. After PBS washes, cells were analyzed using a flow cytometer (Miltenyi Biotec, USA) and the data were analyzed by the Flowing Software 2.5.1 (Turku Bioscience, Finland).

### Western blotting

The cell lysate from each group was prepared using a RIPA lysis buffer containing protease inhibitor. Samples were centrifuged and the total protein concentration of supernatants was determined by Lowry protein assay. Twenty μg of protein was mixed with an equal volume of sample buffer and electrophoresed on SDS–PAGE, then transferred onto PVDF membranes (Santa Cruz, USA). After blocking with 5% skimmed milk, the membrane was washed in PBS with Tween detergent and treated with primary antibodies against the pluripotency markers Oct4 (Abcam, USA) and Sox2 (Abcam, UK), endodermic markers CXCR4 (Santa Cruz, USA), and Sox17 (Santa Cruz, USA), and the internal control β-actin (Santa Cruz Biotechnology, USA) overnight at 4 °C. Primary antibodies against Wnt3a (Santa Cruz, USA) and β-catenin (Santa Cruz, USA) were applied for evaluation of the Wnt signaling pathway. Then, the membrane was exposed to horseradish peroxidase (HRP)-conjugated secondary antibody for 1 h at RT. Finally, the membrane was visualized by luminol reagents (Santa Cruz, USA). The intensity of the bands was quantified using ImageJ software (version 1.41).

### Cell proliferation assay

The proliferation rate was measured using a 5-bromo-2-deoxyuridine (BrdU) assay (Abcam, USA). To this end, cells were seeded at a density of 2 × 10^4^ cells per well of 96-well plates. After completion of the treatment protocol, each well was filled with BrdU reagent before cell harvesting. Cellular DNA was denatured with the fixing solution at RT. After washing with PBS, the anti-BrdU monoclonal antibody was added to each well, and plate was incubated for 1 h at RT. Then, supernatants were discarded and the washing step was repeated followed by the addition of an HRP-conjugated anti-IgG antibody. After the final wash, each well was incubated with a peroxidase substrate and then the reaction was stopped by adding the stop solution when the yellow color was generated. The optical density of the solution was measured using a microplate reader (BDSL Immunoskan, Finland) at 450 nm. Wells containing media alone and seeded cells without BrdU reagent were used as blank and background controls, respectively.

### Acylation assay

To evaluate the total protein acylation, we performed an alkyne-azide cycloaddition click reaction according to a standard protocol [27]. Briefly, cells were seeded onto a 24-well plate at a density of 5 × 10^4^ cell/well and treated as above-mentioned. Medium containing the ω-alkynyl analog of palmitic acid (Alk-C16) was added into the wells at each time point after washing once with PBS and incubated for 24 h at 37 °C with 5% CO_2_ to label the cells. After discarding the medium, cells were then washed with pre-cooled PBS, fixed with − 20 °C pre-chilled methanol. Permeabilization was performed using Triton X-100 at RT for 5 min. Cells were washed with PBS and exposed to a click labeling reagent containing Alexa Fluor 488 Azide (Invitrogen, USA), Tris(2-carboxyethyl) phosphine hydrochloride (TCEP) (Cayman, USA), and CuSO_4_ for 1 h at RT and dark. The wells were then washed with PBS, incubated with 4′,6-diamidino-2-phenylindole (DAPI) for 30 S at RT, and washed three times with PBS. Finally, cells were imaged using the cell imaging system at excitation 488/emission 516 nm for Alexa Fluor488 and excitation 377/emission 477 nm for DAPI.

### Cell cycle analysis

Cell cycle assay was performed using a cell cycle phase determination kit (Cayman Chemical, USA). Cells were seeded on the culture plates according to the manufacturers' instructions. After completion of treatment protocols, the cells were trypsinized, centrifuged, and washed with the assay buffer. The cell pellet was resuspended in the assay buffer and incubated with a fixation buffer. Then, the cell suspension was centrifuged and the supernatant was discarded followed by incubation in staining solution at RT and dark. The percentage of cells in each phase was determined using Flowing software 2.5.1 (Turku Centre for Biotechnology, Finland).

### Statistical analysis

Experimental data are presented as mean ± standard deviation (SD) of the mean from at least three independent experiments. One-way or two-way analysis of variance (ANOVA) followed by Tukey’s post hoc test was applied for groupwise comparisons (GraphPad Software 8.0, USA). A *p* value less than 0.05 was considered to be significant.

## Results

### SCD1 inhibition did not affect cell viability and proliferation

**Fig. 1:**
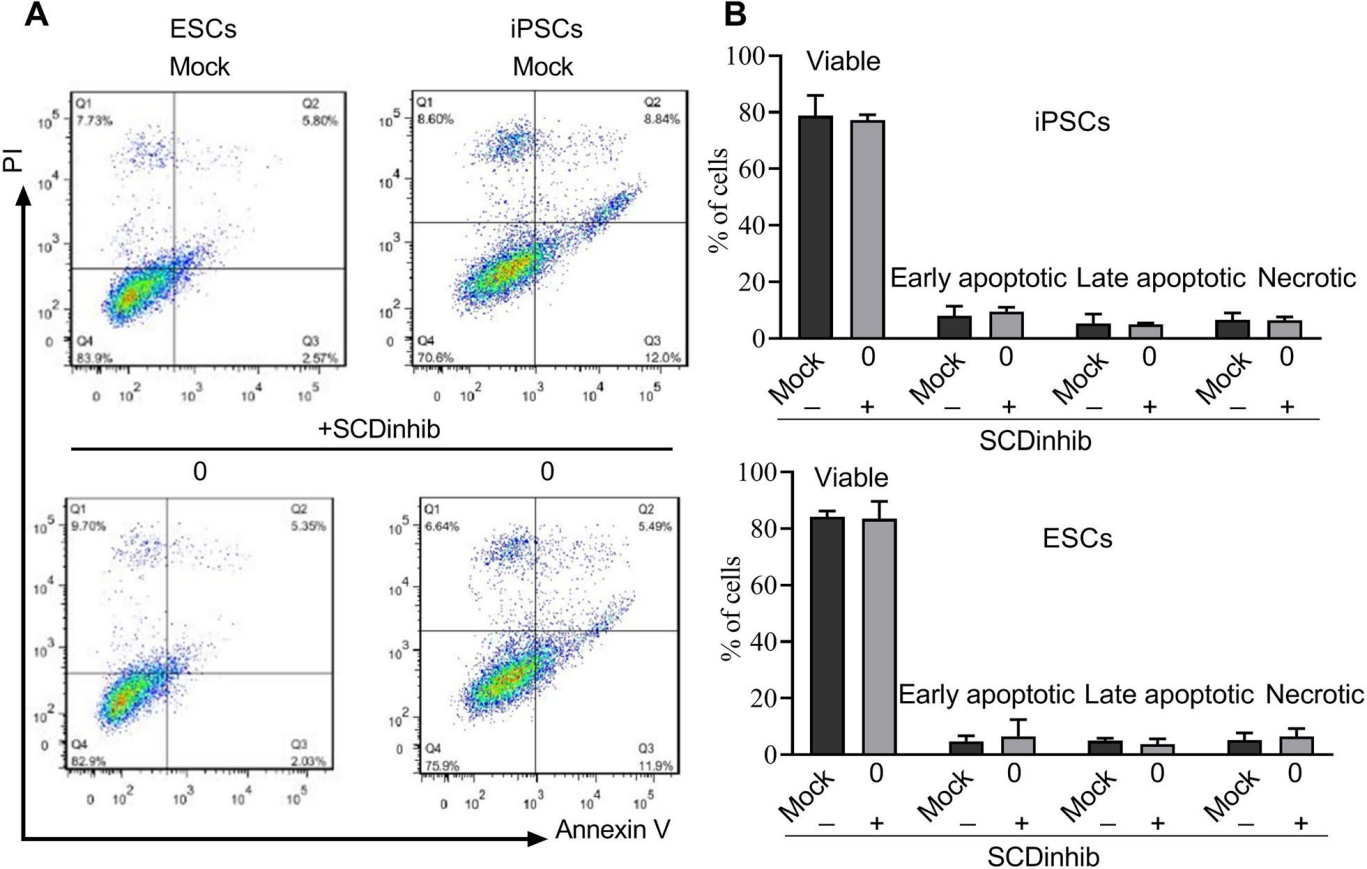
Flow cytometric analysis of pluripotent stem cells after treatment with the SCD1 inhibitor. **A** Representative flow cytometry dot plots showing four-cell fractions: viable cells (annexin V^−^/PI^−^), early apoptotic cells (annexin V^+^/PI^−^), late apoptotic cells (annexin V^+^/PI^+^), and necrotic cells (annexin V^−^/PI^+^). The induced pluripotent stem cells (iPSCs) and embryonic stem cells (ESCs) were mock-treated with DMSO (< 0.05%, above) or treated with 25 nmol/L of SCD1 inhibitor (SCDinhib) on Day 0 of differentiation (below) for 4 days. Cells were then harvested and evaluated for apoptosis and necrosis. **B** Quantification of cells in different groups

**Fig. 2:**
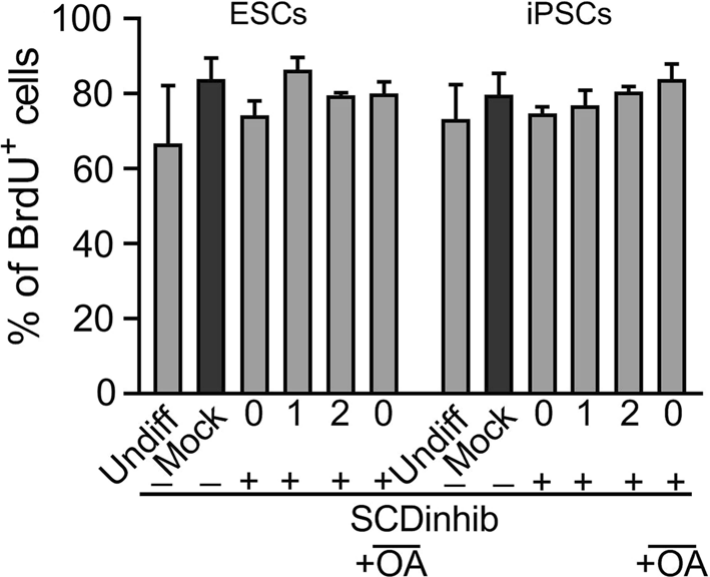
Cell growth determination using BrdU incorporation assay. The induced pluripotent stem cells (iPSCs) and embryonic stem cells (ESCs) were treated with SCD1 inhibitor (SCDinhib) alone at Days 0, 1, or 2 (0, 1, and 2) of differentiation or in combination with oleic acid (OA) on Day 0. Undifferentiated cells (Undiff) and cells treated with DMSO (< 0.05%, Mock) were served as background controls. The cells were harvested on Day 4

In order to evaluate the toxic effect of inhibitor concentration on stem cell viability and proliferation, the hiPSCs and hESCs were treated with the SCD1 inhibitor at Day 0 of endoderm differentiation. The flow cytometry results demonstrated that the applied concentration of the inhibitor did not induce an apoptotic or necrotic effect on cells and the majority of cells were annexin-V and PI negative (Fig. 1). Similarly, the cell proliferation rate, when compared to the mock group, was not altered after inhibition of SCD1 at Days 0, 1, or 2 of endoderm differentiation (*p* > 0.05). According to our data, the proliferation rate was high; more than 70% of cells were BrdU positive at Day 4 of differentiation (Fig. 2).

### SCD1 inhibition maintained stem cell features

**Fig. 3:**
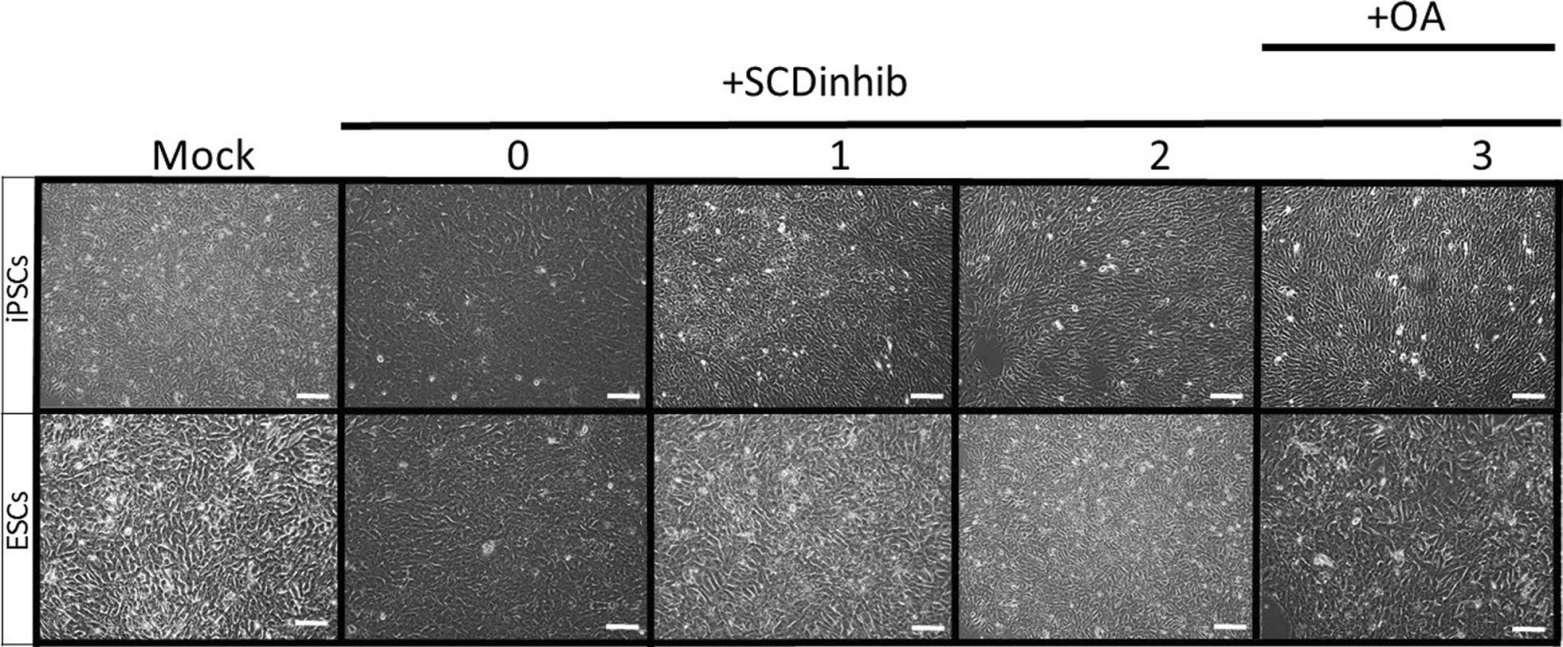
The phase-contrast appearance of differentiated pluripotent stem cells treated with SCD1 inhibitor. SCD1 activity was suppressed at Days 0, 1, or 2 (0, 1, and 2) of endodermal differentiation and imaged at Day 4. Treatment of SCD1 inhibitor at Day 0, but not at Day 1 or 2 or with oleate at Day 0, resulted in maintaining stemness morphology. Scale bar: 100 μm

Bright-field microscopic imaging displayed prominent morphological changes during endoderm induction (Additional file 2: Fig. S1). One day after the differentiation protocol, the cells lost their typical morphological characteristics (i.e., round and compact shapes with high nuclear/cytoplasm ratios) and morphologically different cells appeared. In the next days, the number of morphologically different cells increased. On Day 4, a monolayer of morphologically uniform cells with petal/cobblestone-like morphologies and optically clear cytoplasms were obtained (Additional file 2: Fig. S1). We showed that inhibition of SCD1 only at Day 0 did not change morphology stem cell. It is worth noting that the co-treatment of cells with oleate can change morphological features as appeared with endodermal induction (Fig. 3).

### SCD1 inhibition reduced the expression of endodermal markers

**Fig. 4:**
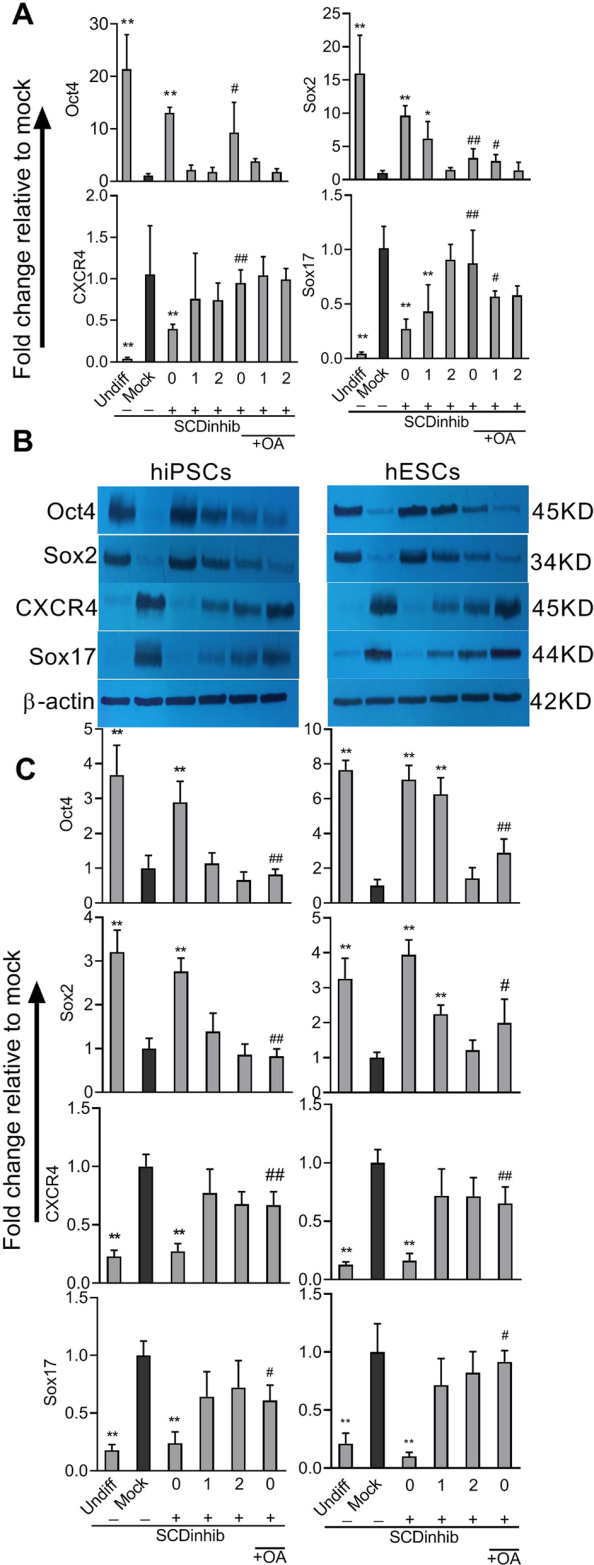
The expression of endoderm and stemness markers following the inhibition of SCD1 activity during induced differentiation. Cells were treated with SCD1 inhibitor (SCDinhib) alone at Days 0, 1, or 2 (0, 1, and 2) of differentiation or in combination with oleic acid (OA) at Day 0. Undifferentiated cells (Undiff) and cells treated with DMSO (< 0.05%, Mock) were served as background controls. The cells were harvested on Day 4. The gene expression in iPSCs (**A**) and protein expression (representative Western blot and quantification) (**B**) of the stemness markers Oct4 and Sox2 and endoderm markers CXCR4 and Sox17 were evaluated in the induced pluripotent stem cells (iPSCs) and embryonic stem cells (ESCs) using quantitative PCR and Western blot, respectively. **C** Quantification of protein expression in different groups. *p < 0.05, **p < 0.01 versus mock, ^#^*p* < 0.05, ^# #^*p* < 0.01 versus the same day in SCDinhib

In differentiated cells, the expression rates of the endodermal markers Sox17 and CXCR4 were robustly elevated, which coincided with the down-regulation of pluripotency markers Oct4 and Sox2 (Fig. 4). These findings indicate the efficiency of the current protocol to commit the cells toward an endoderm-like cell population. To examine the effect of SCD1 inhibition on differentiation of hiPSCs toward endodermal lineage, the one-shot treatment of cells with a non-toxic concentration of SCD1 inhibitor was performed on Day 0, Day 1, and Day 2 of the differentiation process. The alterations of pluripotency and endodermal markers were assessed at both gene and protein levels. When compared to the mock condition, it could be seen that the inhibition of SCD1 significantly prevented the down-regulation of pluripotency marker Oct4 and Sox2 and the increase in endodermal lineage gene markers Sox17 and CXCR4 (Fig. 4). Based on our data, the effect of SCD1 inhibition was smaller on Day 2 of differentiation. As indicated in Fig. 4, in undifferentiated hiPSCs, the expressions of the stemness marker Oct4 were 21.39-fold (gene, *p* < 0.01) and 3.67-fold (protein, *p* < 0.01) whereas these values were 16.01 (*p* < 0.01) and 3.2 (*p* < 0.01) for Sox2. According to our data, both the transcription and protein levels of CXCR4 and Sox17 reached 0.04-fold (gene, *p* < 0.01), 0.22-fold (protein, *p* < 0.01), 0.04-fold (gene, *p* < 0.01), and 0.17-fold (*p* < 0.01) compared to the mock condition.

In differentiated cells, the transcription and protein levels of Oct4 were 13.02-fold (gene, *p* < 0.01) and 2.88-fold (protein, *p* < 0.01) higher than those of the mock, respectively. Notably, when SCD1 was inhibited on Day 0, the values for Sox2 were 9.63-fold (gene, *p* < 0.01) and 2.76-fold (protein, *p* < 0.01) higher compared to the mock condition. We noted that the expressions of stemness markers were not significantly altered when SCD1 was inhibited on Day 1, except for Sox2, which was 6.2-fold (gene, *p* < 0.05) higher in comparison to the mock. In differentiated cells, the expressions of the endoderm marker CXCR4 were 0.39-fold (gene, *p* < 0.01) and 0.27-fold (protein, *p* < 0.01) lower. The expressions of Sox17 were 0.27-fold (gene, *p* < 0.01) and 0.24-fold (protein, *p* < 0.01) lower compared to the mock group when SCD1 was inhibited on Day 0 (Fig. 4). The assessment of endodermal markers revealed that with Day 1 inhibitor the transcription of Sox17 was 0.43-fold (gene, *p* < 0.01) lower than the mock group. The ESCs demonstrated a similar pattern of results after the inhibition of SCD1 during endodermal inhibition. As expected, in the rescue experiment, using oleate together with the SCD1 inhibitor, the endoderm markers efficiently recovered (Fig. 4).

### SCD1 inhibition did not divert differentiation of hiPSCs toward mesodermal or ectodermal lineages

**Fig. 5:**
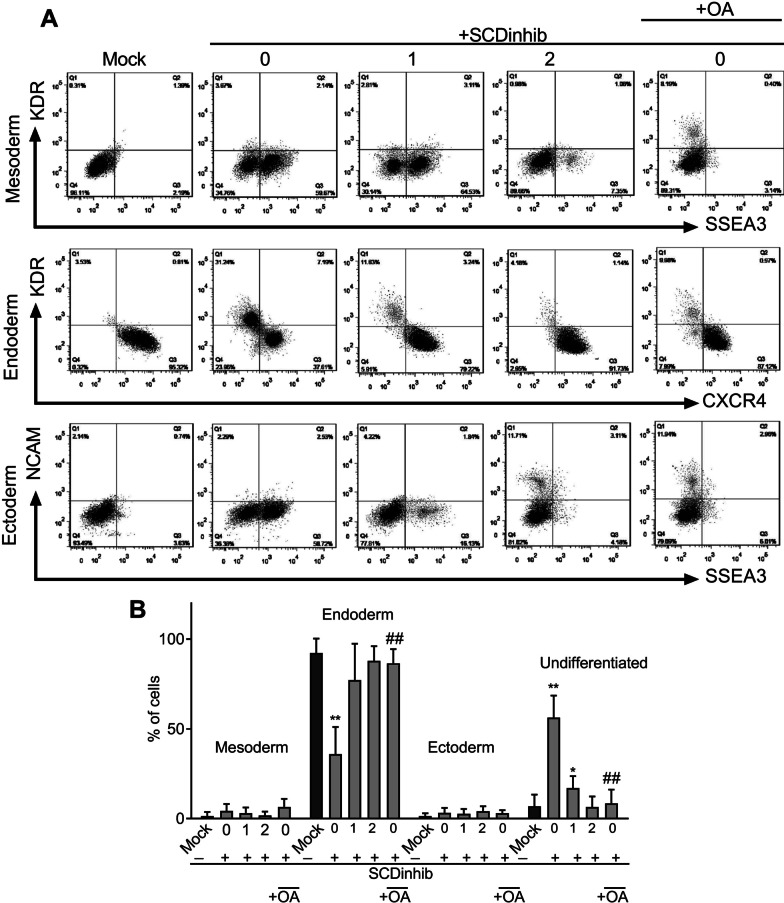
The germ layer markers following inhibition of SCD1 during endodermal differentiation of the induced pluripotent stem cells (iPSCs). Cells were induced to differentiate while treated with SCD1 inhibitor (SCDinhib) alone at Days 0, 1, or 2 (0, 1, and 2) of differentiation or in combination with oleic acid (OA) at Day 0. The cells were harvested on Day 4 and analyzed using flow cytometry. **A** Representative flow cytometry dot plots of the dual staining pattern of mesoderm (KDR^+^, SSEA3^−^), endoderm (CXCR4^+^, KDR^−^), ectoderm (SSEA3^+^, NCAM^+^), and undifferentiated (SSEA3^+^, NCAM^−^) cells. **B** Quantification of flow cytometry results in different groups. *p < 0.05, **p < 0.01 versus mock, ^#^*p* < 0.05, ^# #^*p* < 0.01 versus the same day in SCDinhib

A combination of surface markers, representing the three germ layers cells and undifferentiated cells, was analyzed to distinguish cell types upon SCD1 inhibition and endodermal differentiation. We found that the percentage of endoderm cells decreased 2.56-fold (*p* < 0.01) with the inhibition of SCD1. Interestingly, the percentage of undifferentiated cells in the mock group increased 8.2-fold (*p* < 0.01) and 2.49-fold (*p* < 0.05) following the inhibition of SCD1 on Days 0 and 1, respectively. With the addition of oleate together with SCD1 inhibitor on Day 0, the percentage of endoderm cells increased 2.4-fold (*p* < 0.01) and undifferentiated cells decreased to 6.53-fold (*p* < 0.01), respectively, compared to the same day only with the SCD1 inhibitor. Notably, the inhibition of SCD1 did not significantly alter the percentages of the ectodermal or mesodermal population (Fig. 5).

### SCD1 inhibition resulted in a decrease in total protein acylation

**Fig. 6:**
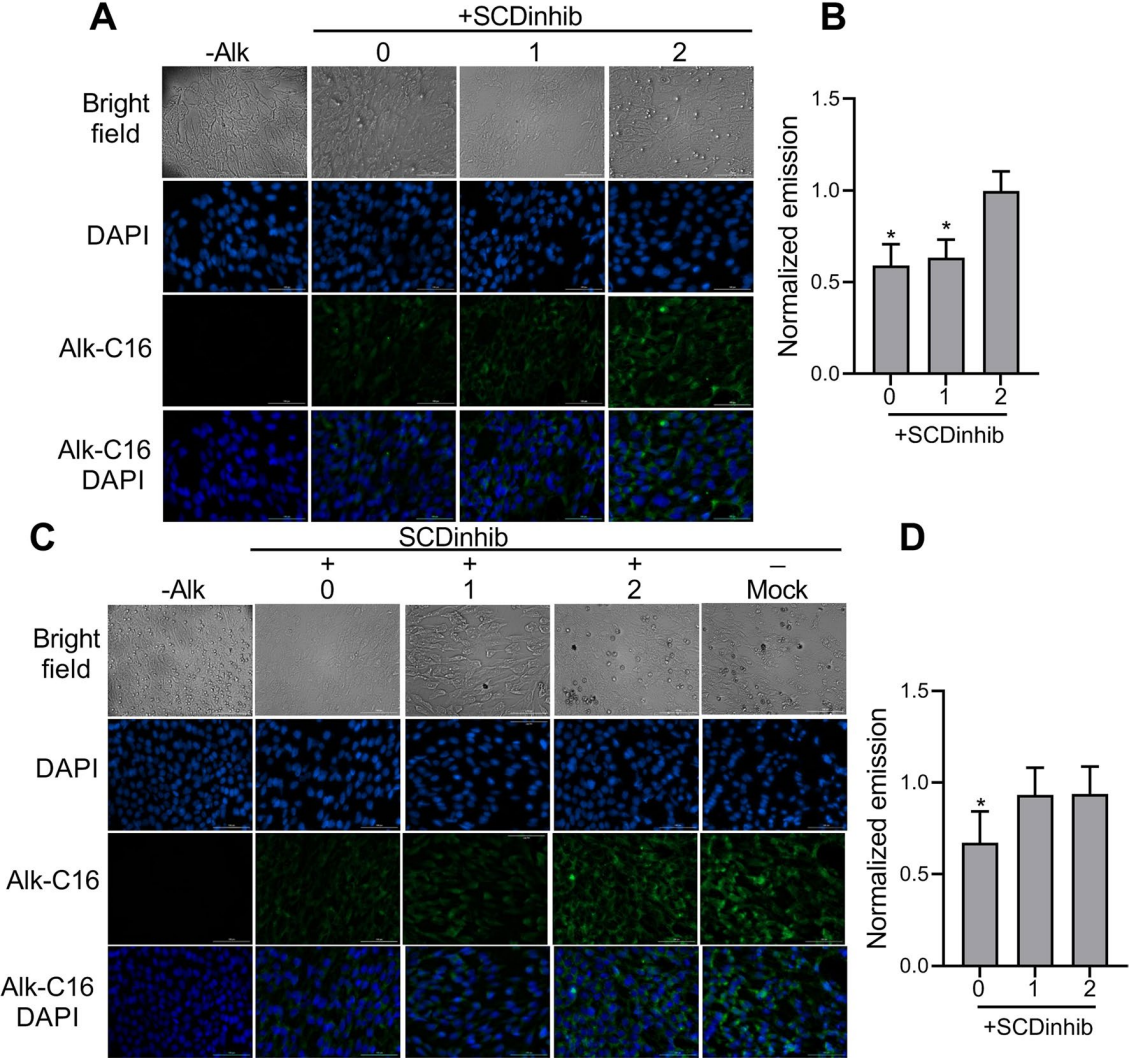
Click chemistry for labeling cellular proteins following inhibition of SCD1 during endoderm differentiation of the pluripotent stem cells. The induced pluripotent stem cells (iPSCs) and embryonic stem cells (ESCs) were induced to differentiate while treated with SCD1 inhibitor (SCDinhib) at Days 0, 1, or 2 (0, 1, and 2). The click reaction was performed 24 h after each treatment. The cells were harvested on Day 4. Representative fluorescence images of iPSCs (**A**) and ESCs (**C**) total protein acylation during endoderm differentiation. Quantification of fluorescence density in iPSCs (**B**) and ESCs (**D**) total protein acylation. The representative image of iPSCs mock condition is shown in Figure S2. *p < 0.05 versus mock. Scale bar: 100 µm

To evaluate the effect of SCD1 inhibition on the acylation of total proteins during endoderm induction, the total protein acylation rate of pluripotent stem cells was measured using click chemistry. Fluorescent imaging results demonstrated a high and constant acylation rate during normal endoderm differentiation (Additional file 3: Fig. S2). However, the inhibition of SCD1 activity at Days 0, 1, or 2 decreased the intensity of the fluorescent emission, which was significant on Day 0. In particular, after the inhibition of SCD1 at Day 0, the emission intensity reached 0.59-fold (*p* < 0.05) and 0.67-fold (*p* < 0.05) lower in the hiPSCs (Fig. 6A, B) and ESCs (Fig. 6C, D), respectively, compared to the mock condition. A decrease in the fluorescent emission was also observed on Day 1 by the inhibition of SCD1 in iPSCs (0.63-fold of mock, *p* < 0.05, Fig. 6A, B).

### SCD1 inhibition increased the percentage of cells in the S phase

**Fig. 7:**
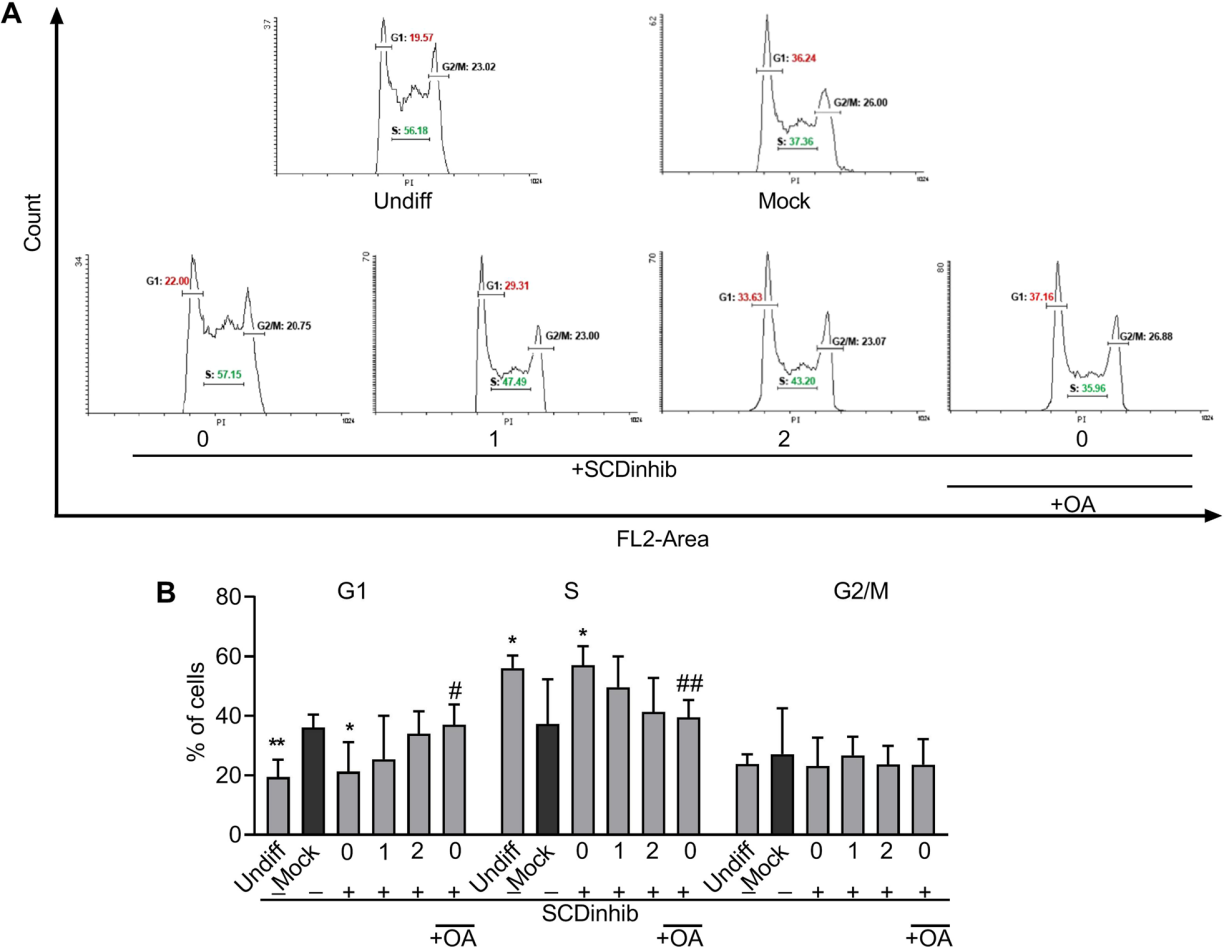
Cell cycle analysis following the inhibition of SCD1 activity during endoderm differentiation. The induced pluripotent stem cells (iPSCs) were treated with SCD1 inhibitor (SCDinhib) alone at Days 0, 1, or 2 (0, 1, and 2) of differentiation or in combination with oleic acid (OA) at Day 0. Undifferentiated cells (Undiff) and cells treated with DMSO (< 0.05%, Mock) were served as background controls. **A** Representative flow cytometers of cells. **B** Quantification of flow cytometry analysis. *p < 0.05, **p < 0.01 versus mock, ^#^*p* < 0.05, ^# #^*p* < 0.01 versus the same Day in SCDinhib

The distributions of differentiated hiPSCs in the cell cycle phases were determined using single color flow cytometry analysis after DNA staining with PI. In the undifferentiated state, the percentages of cells in the G1 and S phases were 19.14 and 56.04, respectively, showing characteristics of PSCs (Fig. 7). In this condition, the percentages of cells in G1 and S phases were 0.53-fold (*p* < 0.01) and 1.5-fold (*p* < 0.05) of the mock group, respectively. The inhibition of SCD1 activity reduced the percentage of cells in the G1 phase (0.58-fold, *p* < 0.05) on Day 0 and concomitantly increased the percentage of cells in the S phase (1.52-fold, *p* < 0.05) as compared to the mock group. In the rescue experiment, the percentages of cells in the G1 and S phases were close to the mock condition. The percentages of cells in the G1 phase increased 1.74 and in the S phases decreased 0.69-fold, respectively, compared to time-matched groups that received SCD1 inhibitor (Fig. 7).

### Inhibition of SCD1 decreased endoderm differentiation through attenuation of the Wnt signaling pathway

**Fig. 8:**
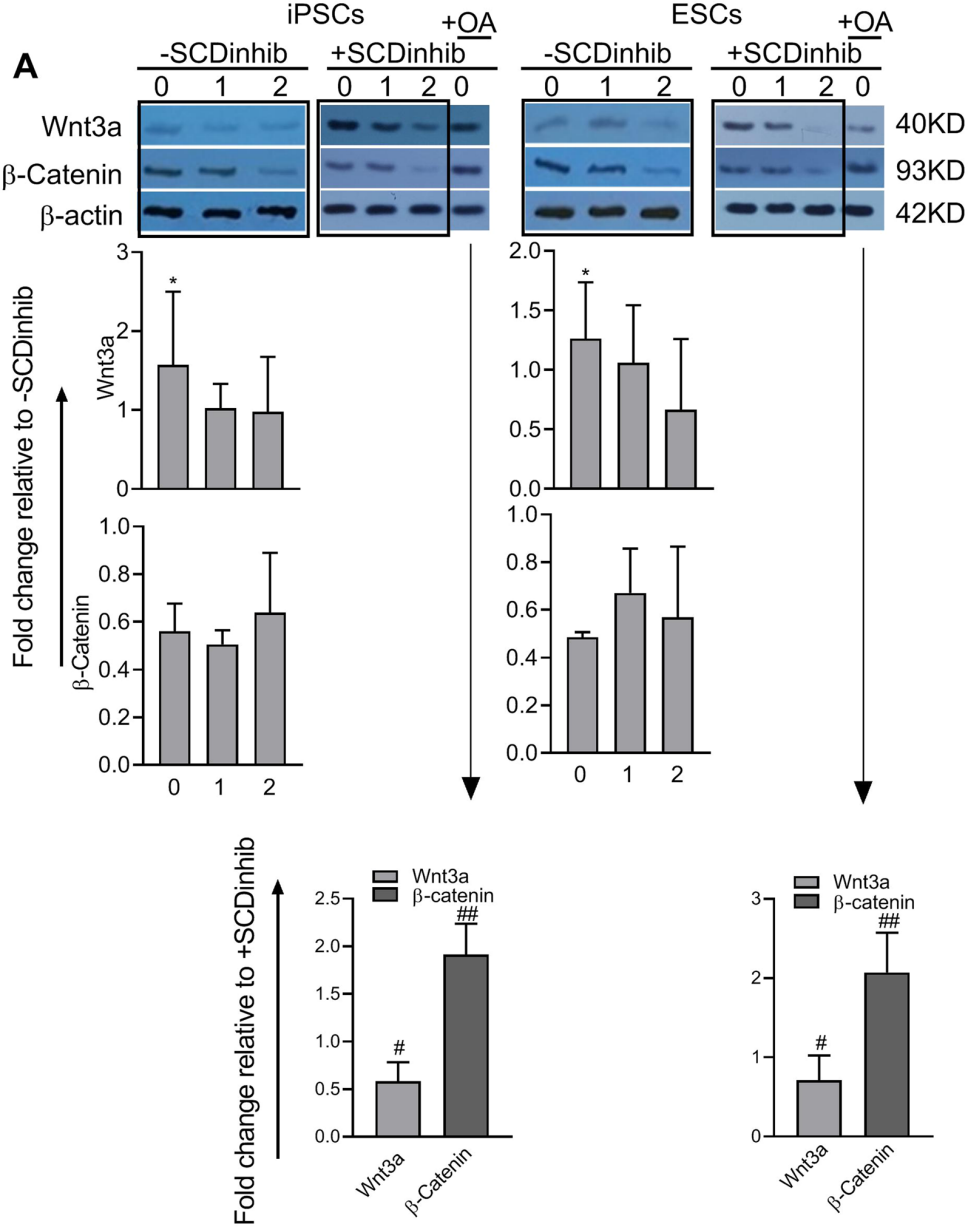
Analysis of the Wnt signaling pathway following endoderm differentiation of pluripotent stem cells. The induced pluripotent stem cells (iPSCs) and embryonic stem cells (ESCs) were treated with SCD1 inhibitor (SCDinhib) alone at Days 0, 1, or 2 (0, 1, and 2) of differentiation or in combination with oleic acid (OA) at Day 0. **A** The representative Western blot and the quantification of Wnt3a and β-catenin. *p < 0.05 versus mock, ^#^*p* < 0.05, ^##^*p* < 0.01 versus the same Day in SCDinhib

Western blotting showed that the protein levels of Wnt3a and its downstream β-catenin gradually decreased from Day 0 to Day 4 of endoderm differentiation (Additional file 4: Fig. S3). The data show a lack of significant difference in the expression of β-catenin following the inhibition of SCD1 on Days 0, 1, or 2 of differentiation. Upon SCD1 inhibition on Day 0 in iPSCs and ESCs, the expression of Wnt3a was 1.57- and 1.26-fold higher than the values observed in the same days without the inhibition of SCD1 (*p* < 0.05), respectively. As expected, the addition of oleate at the same time efficiently counteracted the increasing and decreasing effects of the inhibitor on Wnt3a and β-catenin, respectively (Fig. 8).

## Discussion

PSCs with a high potential to differentiate into adult functional cells are promising options for the treatment of human degenerative diseases [28]. The application of these cells in the clinical setting is mainly hindered by the low differentiation efficiency of in vitro protocols and the possibility of tumorigenicity after transplantation into target organs [29, 30]. In addition, the self-differentiation of these cells during expansion makes the handling of these cells more difficult. The subtle and precise control of differentiation is critical to overcoming these challenges. By increasing our knowledge of the molecular mechanisms governing cell differentiation, we can find an optimum condition for differentiation and maintenance of stem cell behavior in preclinical models or in clinical applications.

MUFAs play important roles in developmental processes through post-translational modification of the proteins involved in differentiation. Thus, understanding the mechanisms by which the metabolism of MUFAs affects stem cell behavior offers promising perspectives to help regulate stem cell differentiation pharmacologically. In the current study, we evaluated the effect of SCD1 inhibition on endodermal differentiation in PSCs by monitoring their protein and transcription levels of molecular markers. We also monitored its effect on total protein acylation, cell viability, cell proliferation, cell cycle, and the Wnt signaling pathway. To assess the specificity of the effects, the main product of SCD1 oleate was co-administrated with the chemical inhibitor.

We observed no significant changes in the PSC proliferation, apoptosis, and necrosis rates after SCD1 inhibition compared to the control group. Therefore, the effects of SCD1 inhibition on PSCs are seemingly independent from cell proliferation, apoptosis, or necrosis. Consistent with our data, the inhibition of SCD1 with similar concentrations of inhibitor did not alter cell viability during liver cell differentiation of hiPSCs [22]. However, treatments of ESC-derived endoderm progenitor cells, ESC-derived hepatocytes, and iPSC-derived cardiomyocytes with the toxic concentration of SCD1 inhibitor were shown to selectively eliminate the cells in undifferentiated states [17]. Notably, the incubation of lung cancer cells with the SCD1 inhibitor-induced toxicity in cancer stem cells [31]. These findings report that the inhibition of SCD1 in different progenitor lineages can yield different outcomes in dynamic growth. The exact mechanisms and underlying machinery participating in this phenomenon are unknown. Our findings showed that the inhibition of SCD1 can reduce the PSC differentiation capacity toward endodermal lineage while maintaining pluripotency of the cells. It confirmed that the efficiency of SCD1 inhibition varied with time during endoderm differentiation. Following the inhibition SCD1 on Day 0 of differentiation, the synthesis of endoderm markers was markedly suppressed. As expected, the treatment of the cells with the SCD1 inhibitor together with exogenous oleate recovered the cells from these effects. The fundamental cellular mechanism that supports these effects is that palmitoleate, like oleate, is also the product of SCD1. It seems that oleate alone can compensate for the deficiency of SCD1 activity during endodermal differentiation of PSCs. In line with our study, the suppression of SCD1 activity during hepatic differentiation delayed the production of hepatic function markers during liver cell maturation [22]. Presumably, the activity of SCD1 is reduced concomitantly by the progression of cells toward specialized cells [22]. Data further confirmed the pivotal role of SCD1 in the development of the liver in rat embryos [23]. The inhibition of SCD1 in an early phase of pregnancy led to severe defects in fetal liver development indicated by the down-regulation of HNF1α, AFP, ALB, and CYP450 [23]. These effects were blunted in the presence of oleate [22, 23]. It was suggested that using IWP-2 to inhibit Porcn, an acyltransferase catalyzing the addition of MUFAs onto Wnts, reduced the expression of endoderm markers such as Sox17 and FOXa2 a few hours before initiation of endoderm induction [32]. Along with our results, it can be proposed that SCD1 and its products are involved in the development and differentiation of stem cells into the endodermal lineages [33, 34]. In contrast, some reports demonstrated different outcomes associated with the function of SCD1 products in stem cells. It was recently found that the treatment of MEFs during their reprogramming into iPSCs resulted in iPSCs that formed more colonies in comparison to the non-treated control. Furthermore, these iPSCs had a higher capacity for palmitoleate and oleate synthesis. Remarkably, the inhibition of Porcn can lead to a reduction in the pluripotency of the ESCs [35]. These results indicate that SCD1 products can act in a context-dependent manner in terms of cell fate acquisition. The function of SCD1 products can promote cell differentiation and are critical in the early phase of differentiation. Meanwhile, they can help to maintain stem cell features. Similar effects have been reported in molecules dependent on MUFAs, such as Wnt molecules. Several conflicting reports in the literature show that Wnt molecules promote either stemness or differentiation and the lineage specification of stem cells [36–40].

To investigate the possible role of SCD1 in the proteins acylation rate, we used click chemistry and fluorescent imaging during endoderm differentiation and following the SCD1 inhibition. An Alk-C16 probe analysis showed that endoderm differentiation is associated with a significant protein acylation signal. This finding implies that a great deal of proteins undergo acylation by fatty acids in stem cells subjected to endodermal differentiation. Previously, it has been shown that Alk-C16 can be converted into its monounsaturated form Alk-C16:1 by the activity of SCD1 [41]. Therefore, the intensity of the signal is associated with the incorporation of both saturated and monounsaturated forms of the probe. Notably, the acylation rate significantly decreased when SCD1 was inhibited on Day 0. We interpreted this event as correlating with the reduction of the monounsaturated form of Alk-C16 for incorporation and biochemical reactions.

Previous studies showed the significant role of Activin A and Wnt/β-catenin pathways in the endodermal differentiation of ESCs [32, 42, 43]. Activin A-induced SCD1 can produce metabolites that can post-translationally modify the Wnt molecules [43]. The contribution of SCD1 in the activity of Wnt signaling was assessed using western blotting and gene expression analysis. Our results showed mild to moderate suppression of Wnt3a/β-catenin during the differentiation of iPSCs toward endodermal lineages, indicating the crucial role of SCD1 in early-stage stem cell differentiation. Following the treatment of cells with the SCD1 inhibitor, the expression of Wnt was increased with non-significant changes in β-catenin. One reason for the Wnt induction may be that its expression is a compensatory response after the suppression of MUFA acylation via the SCD1 product. Additionally, the slight-to-mild suppression of β-catenin can be related to the attenuation of the Wnt signaling pathway following SCD1 inhibition, as these features result in the reduced endoderm differentiation of iPSCs. Based on these results, we hypothesize that the changes in the Wnt/β-catenin signaling pathway are possibly due to reduced MUFA acylation. Moreover, it has been noted that SCD1 activity can be changed during cell differentiation. Evaluation of other MUFAs with respect to different types of Wnt molecules such as Wnt5a will enable us to understand the role of MUFA acylation in diverse differentiation process. MUFAs are much more essential in the first stages of differentiation than in the latter steps, indicating that the immediate triggering activity of SCD1 is essential for endodermal differentiation in hiPSCs. As indicated previously, the generation of the primitive streak and subsequently formation of mesendodermal cells are a prerequisite for the formation of endoderm [26, 44]. Most probably, MUFAs are required for the production of primitive streak- and mesendoderm-like cells; however, the hypothesis needs to be investigated in future studies.

## Conclusion

The dynamics of SCD1 activity are crucial to the early commitment and differentiation of hiPSCs to endoderm lineage. We showed that SCD1 inhibition attenuates the Wnt/β-catenin signaling pathway, conferring the maintenance of hiPSCs by opposing the initiation of endoderm differentiation. The requirement for SCD1 activity in the endoderm commitment of PSCs may be of importance in disorders of endoderm-derived organs and dysregulated metabolism.

## Supplementary Information

**Additional file 1: Table S1.** List of primers used for gene expression analysis in this study.

**Additional file 2: Fig. S1.** The phase-contrast appearance of differentiating pluripotent stem cells. The induced pluripotent stem cells (iPSCs) and embryonic stem cells (ESCs) were differentiated toward endoderm lineage with activin A and defined FBS. Cells exhibited typical morphology of pluripotent stem cells on Day 0. Over differentiation, a loss of typical stem cell morphology was noted on Day 2. Very different cellular morphologies appeared in the following days until at Day 4 a monolayer of morphologically uniform cells was obtained. Scale bar: 100 μm.

**Additional file 3: Fig. S2.** Click chemistry for imaging of whole protein acylation during endoderm differentiation of the induced pluripotent stem cells (iPSCs). Cells were induced to differentiate and imaged with 24 h intervals of 0 to 4 days (0, 1, 2, 3, and 4). **A** Cells were treated with Alk-C16 before reaction with Alexa Fluor 488 azide (green). Nuclei were stained with DAPI (blue) to normalize for the cell number. **B** Quantification of fluorescence density. Alk-C16; ω-alkyne palmitic acid. Scale bar: 100 µm.

**Additional file 4: Fig. S3.** Analysis of the Wnt signaling pathway during endoderm differentiation of pluripotent stem cells. The induced pluripotent stem cells (iPSCs) and embryonic stem cells (ESCs) were induced to differentiate and analyzed at Days 0, 1, 2, 3, and 4 (0, 1, 2, 3, and 4). The cells were harvested on Day 4. **A** The representative Western blot of Wnt3a and β-catenin. **B** Quantification of protein expression in different groups. *p < 0.05 and **p < 0.01 versus Day 0, ^#^*p* < 0.05, ^##^*p* < 0.01 versus Day 1, ^$^*p* < 0.05, ^$$^*p* < 0.01 versus Day 2, ^@^*p* < 0.05, ^@@^*p* < 0.01 versus Day 3.

## Data Availability

Not applicable.

## Abbreviations

Alk-C16: ω-Alkynyl analog of palmitic acid
b-FGF: Basic human recombinant FGF
BrdU: 5-Bromo-2-deoxyuridine
D-FBS: Defined-FBS
FBS: Fetal bovine serum
hESCs: Human embryonic stem cells
hiPSCs: Human-induced pluripotent stem cells
miPSCs: Mouse-induced pluripotent stem cells
MUFAs: Monounsaturated fatty acids
PBS: Phosphate-buffered saline
SCD1: Stearoyl-coenzyme A desaturase 1
SD: Standard deviation
SFAs: Saturated fatty acids
TCEP: Tris(2-carboxyethyl) phosphine hydrochloride
