# Supplementary material for: A small molecule modulating monounsaturated fatty acids and Wnt signaling confers maintenance to induced pluripotent stem cells against endodermal differentiation
Source: Stem Cell Res Ther. 2021 Oct 21;12:550. doi: 10.1186/s13287-021-02617-x (PMC8532309; doi:10.1186/s13287-021-02617-x)
Supplement: Supplementary file 1 — Additional file 1: Table S1. List of primers used for gene expression analysis in this study. [file 13287_2021_2617_MOESM1_ESM.docx]

Supplementary Table 1. List of primers used for gene expression analysis in this study

| Gene | Forward primer (5ʹ-3ʹ) | Reverse primer (5ʹ-3ʹ) | Accession No. |
| --- | --- | --- | --- |
| Oct4 | GAAGGATGTGGTCCGAGTG | ATCCTCTCGTTGTGCATAGTC | NM_002701.6 |
| Sox2 | GGGAAATGGGAGGGGTGCAAAAGAGG | TTGCGTGAGTGTGGATGGGATTGGTG | [NM_003106.4](https://www.ncbi.nlm.nih.gov/entrez/viewer.fcgi?db=nucleotide&id=1519313016) |
| CXCR4 | GACCGCTTCTACCCCAATGA | GCAATAGCAGGACAGGATGAC | [NM_001348056.2](https://www.ncbi.nlm.nih.gov/entrez/viewer.fcgi?db=nucleotide&id=1677501180) |
| Sox17 | GGACCGCACGGAATTTGAACA | GCTGTCGGGGAGATTCACAC | [NM_022454.4](https://www.ncbi.nlm.nih.gov/entrez/viewer.fcgi?db=nucleotide&id=1519243972) |
| GAPDH | CTGACTTCAACAGCGACACC | CGTTGTCATACCAGGAAATGAGC | [NM_001357943.2](https://www.ncbi.nlm.nih.gov/entrez/viewer.fcgi?db=nucleotide&id=1676440496) |
