# Supplementary figures and images for: A small molecule modulating monounsaturated fatty acids and Wnt signaling confers maintenance to induced pluripotent stem cells against endodermal differentiation
Source: Stem Cell Res Ther. 2021 Oct 21;12:550. doi: 10.1186/s13287-021-02617-x (PMC8532309; doi:10.1186/s13287-021-02617-x)

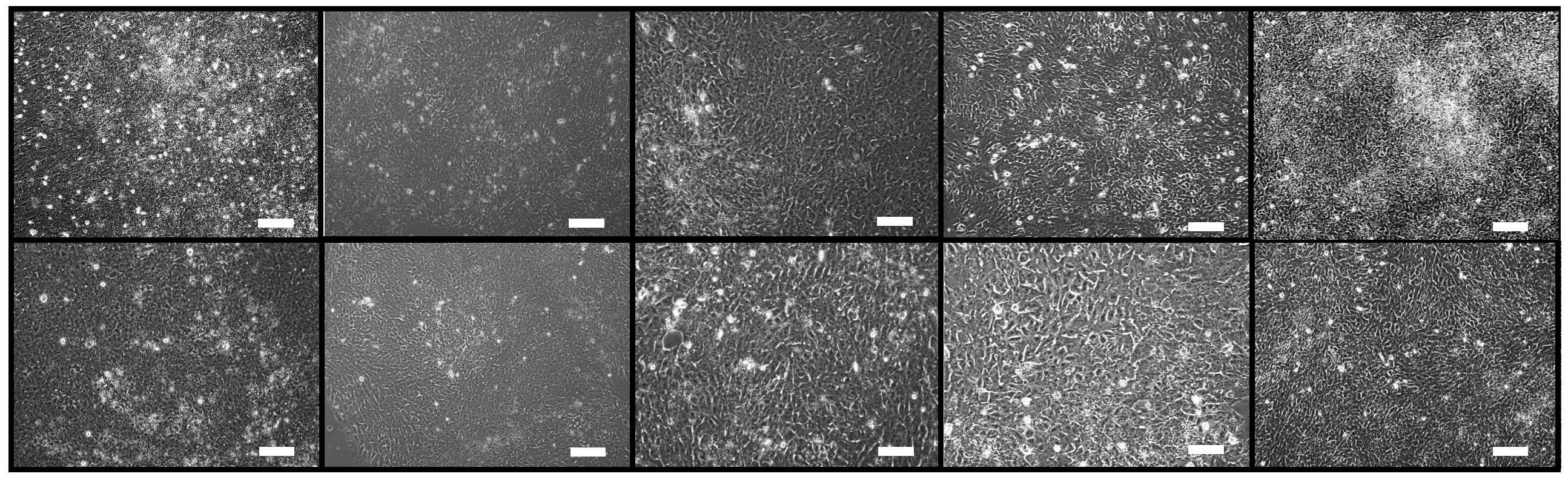

Supplement: Supplementary file 2 — Additional file 2: Fig. S1. The phase-contrast appearance of differentiating pluripotent stem cells. The induced pluripotent stem cells (iPSCs) and embryonic stem cells (ESCs) were differentiated toward endoderm lineage with activin A and defined FBS. Cells exhibited typical morphology of pluripotent stem cells on Day 0. Over differentiation, a loss of typical stem cell morphology was noted on Day 2. Very different cellular morphologies appeared in the following days until at Day 4 a monolayer of morphologically uniform cells was obtained. Scale bar: 100 μm. [file 13287_2021_2617_MOESM2_ESM.jpg]

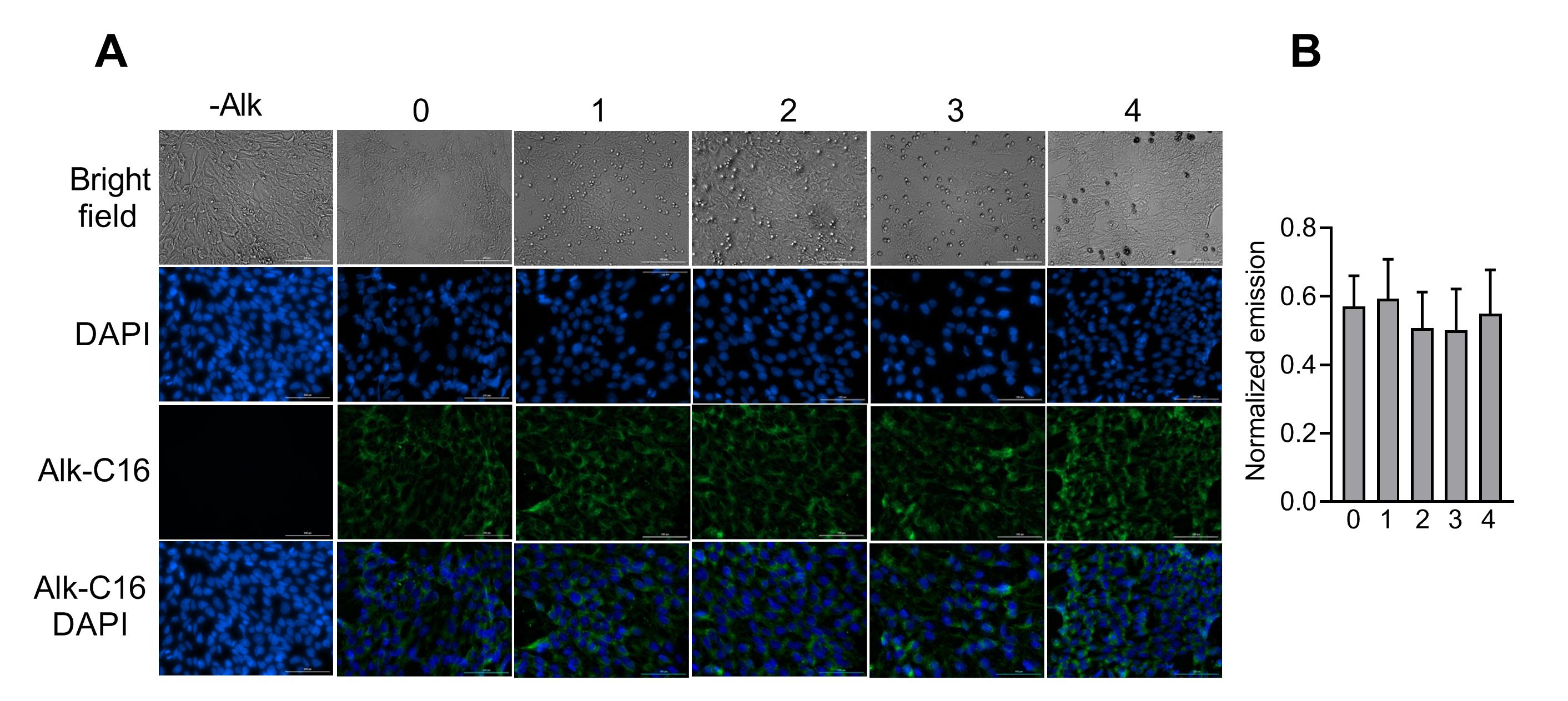

Supplement: Supplementary file 3 — Additional file 3: Fig. S2. Click chemistry for imaging of whole protein acylation during endoderm differentiation of the induced pluripotent stem cells (iPSCs). Cells were induced to differentiate and imaged with 24 h intervals of 0 to 4 days (0, 1, 2, 3, and 4). A Cells were treated with Alk-C16 before reaction with Alexa Fluor 488 azide (green). Nuclei were stained with DAPI (blue) to normalize for the cell number. B Quantification of fluorescence density. Alk-C16; ω-alkyne palmitic acid. Scale bar: 100 µm. [file 13287_2021_2617_MOESM3_ESM.jpg]

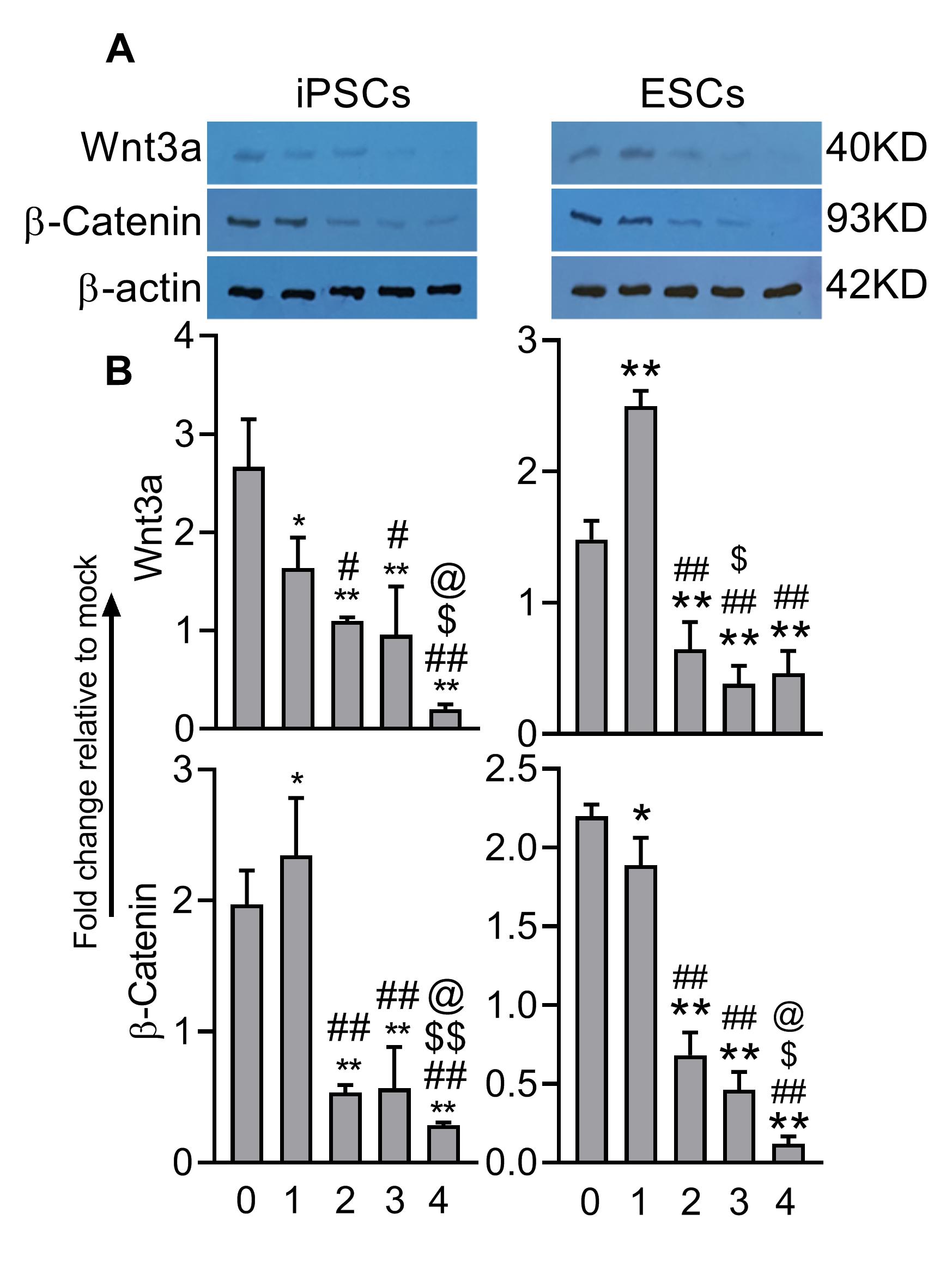

Supplement: Supplementary file 4 — Additional file 4: Fig. S3. Analysis of the Wnt signaling pathway during endoderm differentiation of pluripotent stem cells. The induced pluripotent stem cells (iPSCs) and embryonic stem cells (ESCs) were induced to differentiate and analyzed at Days 0, 1, 2, 3, and 4 (0, 1, 2, 3, and 4). The cells were harvested on Day 4. A The representative Western blot of Wnt3a and β-catenin. B Quantification of protein expression in different groups. *p < 0.05 and **p < 0.01 versus Day 0, #p < 0.05, ##p < 0.01 versus Day 1, $p < 0.05, $$p < 0.01 versus Day 2, @p < 0.05, @@p < 0.01 versus Day 3. [file 13287_2021_2617_MOESM4_ESM.jpg]
